# Editorial: Positive psychology in health management

**DOI:** 10.3389/fpsyg.2023.1334314

**Published:** 2023-11-23

**Authors:** Anni Wang, Feifei Huang, Yufang Guo, Fang Lei, Yuting Song

**Affiliations:** ^1^School of Nursing, Fudan University, Shanghai, China; ^2^School of Nursing, Fujian Medical University, Fuzhou, China; ^3^School of Nursing and Rehabilitation, Shandong University, Jinan, China; ^4^School of Nursing, University of Minnesota Twin Cities, Minneapolis, MN, United States; ^5^School of Nursing, Qingdao University, Qingdao, China

**Keywords:** positive psychology, health management, resilience, workforce, intervention

Positive psychology aims to understand how people can better their lives, and ultimately, flourish. Since Martin Seligman spearheaded this movement, focusing on the positives in life rather than focusing on the negatives, substantial research has been conducted. This research indicates that positive psychological resources, including resilience, self-efficacy, optimism, hope, self-efficacy, and self-esteem, can assist individuals in developing their personal capabilities. These capabilities are associated with higher levels of physical, psychological, social, and behavioral wellbeing (Seligman, 2019).

Health management is a broad topic encompassing various aspects of health-related issues, involving policy-making, legal regulation, workforce management, healthcare quality, and specific disease management. There has been a noticeable increase in research applying positive psychology to health management (Rusk and Waters, 2013). The growing body of theoretical and empirical studies demonstrates that positive psychological theories have positive effects on the patients' physical and mental health, quality of life, as well as on healthcare workers' job satisfaction, burnout, and quality of work life (Rusk and Waters, 2013; Goyal et al., 2014; Gruman and Budworth, 2022). Over the past decade, disease management and health workforce management have emerged as two major subjects extensively investigated in the context of positive psychology (Aspinwall and Tedeschi, 2010). By integrating positive psychology and health management together, this is field seeks to foster cutting-edge ideas and research to explore multidisciplinary approaches of positive psychology in disease management and health workforce management.

This call for submissions on *Positive psychology and health psychology* has received an excellent response. The Research Topic comprises 16 studies involving over 10 thousands participants, which have garnered a total of 26,525 total views and 7,102 downloads worldwide until drafting this editorial. The studies encompass a diverse range of populations, including patients with conditions such as breast cancer (Ma, Wan et al.), acute leukemia (Peng et al.), and COVID (Zheng et al.), as well as those living with HIV (Orth and Van Wyk) and schizophrenia (Dong et al.). Additionally, it includes studies on populations facing stress, such as general populations (Burke and Dunne), empty nesters (Song et al.), and employees (Bala Subramanian et al.). Furthermore, the Research Topic addresses health providers, including family doctors (Sun et al.), health professionals (Wang et al.) nurses (Guo et al.), and emergency physicians (Xu et al.). It also encompasses studies on various student populations, including college students (Liu et al.; Ma, Li et al.), university students (Chen et al.), and nursing students (Huang et al.).The findings derived from the current Research Topic align closely with the aims outlined this topic:

1) They demonstrate how the construct of positive psychology mitigates negative consequences for patients and family coping with disease burden. For instance, post-traumatic growth is positively correlated with social support (Ma, Wan et al.). Additionally, the pillars of Lifestyle medicine serve as predictors of psychological flourishing (Burke and Dunne), while resilience mediates mental health literacy and positive coping styles (Song et al.).
2) They also illustrate how the construct of positive psychology mitigates the negative consequences for healthcare workers experiencing job-related stress. This includes the mediating effect of culture on positive leadership and quality of work and life (Sun et al.), as well as individual and organizational resilience acting as predictor of job performance (Wang et al.). Furthermore, psychological capital serve as a predictor of anxiety and depression (Xu et al.), and gratitude mediates distributive justice and organizational citizenship behaviors (Bala Subramanian et al.).
3) The findings demonstrate how positive psychology helps to mitigate the negative consequences of learning stress. This encompasses emotion regulation and positive psychological capital as predictors of depression, with stress beliefs and core self-evaluations of procrastination mediating these effects (Liu et al.; Ma, Li et al.). Additionally, learning motivation and target positioning on emotion regulation and happiness (Chen et al.), while resilience and positive coping style affect the relationship between maladaptive perfectionism and academic procrastination (Huang et al.).
4) They also explore the efficacy, effectiveness and implementation of positive psychology-based intervention for patients or healthcare workers to promote resiliency. This includes cognitive intervention intervention for improving quality of life (Peng et al.), online expressive writing for reducing psychological distress (Zheng et al.), WeChat-based self-compassion training to enhance the treatment adherence (Dong et al.), and a nurse-manager dualistic intervention program to alleviate burnout (Guo et al.).
5) Finally, the findings contribute to the development of culturally sensitive and contextually innovative theories and instruments. Notably, they aid in the conceptualizing of mental wellness among adolescents living with HIV (Orth and Van Wyk). These findings hold significant potential for advancing future theoretical and empirical studies in this field.

However, several limitations should be emphasized here. First, a majority of the articles employed an observational cross-sectional design, primarily focusing on examining mediation or moderation effects. Consequently, the full potential of positive constructs in enhancing long-term adaptation outcomes under stress remains incompletely elucidated (Lam et al., 2012). Secondly, one of our aims was to discern distinct adaptation trajectories under stress, where positive psychological resources could exert an effect. Regrettably, this goal was realized in the current Research Topic. Third, there is a notable scarcity of articles dedicated to theory and instrument development. Future research endeavors should be directed toward formulating novel theories and instruments to delve deeper into the construct within positive psychology (Southwick et al., 2014). Fourthly, the examination of the efficacy, sustainability and implementation challenges of complex intervention programs targeting various health-related stressful population should be the next stride in advancing our understanding (Goyal et al., 2014; Skivington et al., 2021).

## Author contributions

AW: Writing – original draft, Writing – review & editing. FH: Writing – review & editing. YG: Writing – review & editing. FL: Writing – review & editing. YS: Writing – review & editing.
